# A Pilot Study Evaluating the Safety of Intravenously Administered Human Amnion Epithelial Cells for the Treatment of Hepatic Fibrosis

**DOI:** 10.3389/fphar.2017.00549

**Published:** 2017-08-23

**Authors:** Rebecca Lim, Alexander Hodge, Gregory Moore, Euan M. Wallace, William Sievert

**Affiliations:** ^1^The Ritchie Centre, Hudson Institute of Medical Research, Clayton VIC, Australia; ^2^Department of Obstetrics and Gynaecology, Monash University, Melbourne VIC, Australia; ^3^Centre for Inflammatory Diseases, Monash University, Melbourne VIC, Australia; ^4^Department of Gastroenterology and Hepatology, Monash Health, Melbourne VIC, Australia

**Keywords:** liver cirrhosis, phase 1 clinical trial, safety trial, stem cells, cell therapy

## Abstract

Liver cirrhosis is the 6th leading cause of death in adults aged 15–59 years in high-income countries. For many who progress to cirrhosis, the only prospect for survival is liver transplantation. While there is some indication that mesenchymal stem cells may be useful in reversing established liver fibrosis, there are limitations to their widespread use – namely their rarity, the need for extensive serial passaging and the associated potential for genomic instability and cellular senescence. To this end, we propose the use of allogeneic amnion epithelial cells. This clinical trial will assess the safety of intravenously delivered allogeneic human amnion epithelial cells (hAECs) in patients with compensated liver cirrhosis. This will also provide clinical data that will inform phases 2 and 3 clinical trials with the ultimate goal of developing hAECs as a therapeutic option for patients with cirrhosis who are at significant risk of disease progression. We will recruit 12 patients with compensated cirrhosis, based on their hepatic venous pressure gradient, for a dose escalation study. Patients will be closely monitored in the first 24 h post-infusion, then via daily telephone interviews until clinical assessment on day 5. Long term follow up will include standard liver tests, transient elastography and hepatic ultrasound. Ethics approval was obtained from Monash Health for this trial 16052A, “A Pilot Study Evaluating the Safety of Intravenously Administered Human Amnion Epithelial Cells for the Treatment of Liver Fibrosis, A First in Adult Human Study.” The trial will be conducted in accordance to Monash Health Human Ethics guidelines. Outcomes from this study will be disseminated in the form of conference presentations and submission to a peer reviewed journal. This trial has been registered on the Australian and New Zealand Clinical Trials Registry ACTRN12616000437460.

## Introduction

The World Health Organization has identified cirrhosis as the 6th leading cause of death in adults aged 15–59 years in high-income countries and the 9th most common cause of death in that age group in low-income countries (Lopez et al., 2006). Liver fibrosis results from a complex wound healing activity that involves increased extracellular matrix (ECM) synthesis as well as replacement of apoptotic or necrotic hepatocytes. Cirrhosis results from persistent and unregulated hepatic wound healing in response to ongoing hepatocyte injury. While previously considered as a static and irreversible outcome of progressive fibrosis leading invariably to death, we now know that this process is dynamic and potentially reversible for many patients. Extensive clinical data shows that liver fibrosis can resolve following adequate treatment or resolution of the injury stimulus, such as long term viral suppression in hepatitis B patients (Marcellin et al., 2013).

For many patients who progress to cirrhosis, a lack of response to existing therapy or the absence of effective treatment means that their only prospect for survival is liver transplantation. As the demand for liver donors continues to increase, there is a clear need for alternatives to whole organ transplantation for these patients. The use of stem cells for liver fibrosis has been previously proposed, based on preclinical evidence that mesenchymal stem cells (MSCs) ameliorate liver fibrosis in experimental animal models of the disease (Tsai et al., 2009; Wang et al., 2012; Meier et al., 2015). The postulated mechanisms of action include polarization of macrophages to an anti-fibrotic phenotype, reversal of activated stellate cells to a quiescent state (Chen et al., 2017) and general mitigation of hepatic inflammation.

To date, there are 56 clinical trials registered on ClinicalTrials.gov assessing the safety and efficacy of MSCs in liver cirrhosis. These include primary biliary cirrhosis, as well as liver cirrhosis associated with alcoholic liver disease and viral hepatitis. The MSCs used in these trials include both autologous and allogeneic sources, from adipose tissue, bone marrow, umbilical cord, and menstrual blood. A Korean study has reported promising results in a phase II trial where hepatic arterial injection of autologous bone marrow derived MSCs were reported to improve liver fibrosis (Suk et al., 2016). While such an outcome is promising, there remain many more trials whose outcomes are yet to be reported and may inform the future utility of MSCs for liver cirrhosis. Additionally, there are limitations to using MSCs associated with their rarity and their need for extensive passaging. These include the potential for genomic instability (Wang et al., 2013) and cellular senescence (Schellenberg et al., 2011) seen with passaged MSCs and as such, we have ventured into alternatives such as human amnion epithelial cells (hAECs), which have similarly potent anti-inflammatory and anti-fibrotic properties (Manuelpillai et al., 2010, 2012; Murphy et al., 2010a, 2011; Hodges et al., 2012; Hodge et al., 2014; Tan et al., 2016). Specifically, hAECs appear to influence inflammation and fibrosis by interacting with a multitude of cell types including fibroblasts (Vosdoganes et al., 2012), neutrophils (Tan et al., 2016), and macrophages (Manuelpillai et al., 2012; Tan et al., 2014). These outcomes appear to be independent of significant cell engraftment, but are instead indicative of a paracrine effect, similar to what has been previously reported in MSCs. Considering the complex cellular interactions involved in hepatic wound healing, hAECs may provide a multi-targeted approach needed for effective collagen degradation and hepatocyte regeneration. hAECs are isolated from the placenta, a widely available and non-controversial source. These fetal stem cells do not elicit immune-mediated rejection by the host and have intrinsic immune modulating characteristics that can induce hepatic fibrosis regression and stimulate liver regeneration. A clinically compliant method of hAEC isolation was previously established with a view to developing cell-based therapy (Murphy et al., 2010b). The hAECs are being used in a phase 1b trial, in babies with established bronchopulmonary dysplasia. As of the preparation of this manuscript, five babies have received allogeneic hAECs with no safety concerns (ACTRN12614000174684).

The objective of this phase 1 trial is to primarily assess the safety of intravenously delivered allogeneic hAECs in patients with compensated liver cirrhosis. This open label study will also provide clinical data that will inform phases 2 and 3 clinical trials with the ultimate goal of developing hAECs as a therapeutic option for patients with cirrhosis who are at significant risk of disease progression. For patients who progress to decompensated cirrhosis, the only effective therapeutic option is whole organ replacement. Liver transplantation is an established life-saving treatment for patients with end-stage liver disease; however, success depends on donor graft availability and recipients require life-long immunosuppression. In contrast, hAEC based therapy has the potential to prevent progression of cirrhosis and thus the need for transplantation.

## Methods and Analysis

### Study Design

**FIGURE 1:**
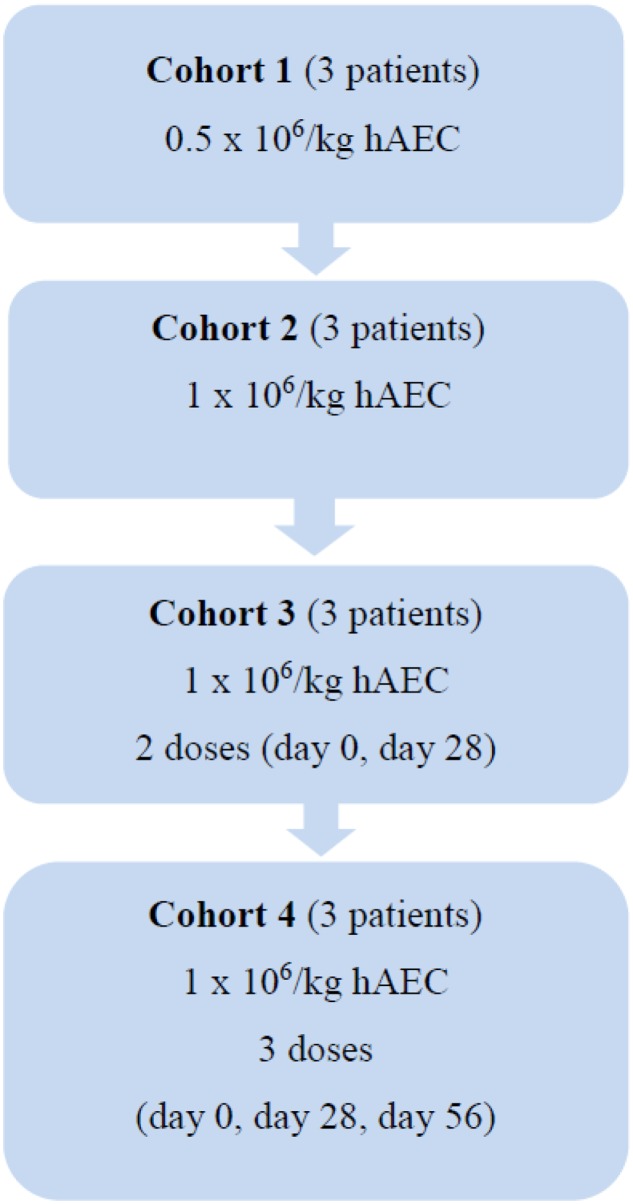
Studydesign.

In this open label phase 1 study, the safety and tolerability of hAEC transplantation will be examined in 12 patients with compensated cirrhosis (**Figure 1**). They will be divided into four cohorts. Each cohort will include three patients treated sequentially (patient 1, then patient 2, then patient 3). Each patient will be observed for serious adverse events (SAEs) for 5 days post intravenous (IV) hAEC infusion before the next patient is enrolled. In Cohort 1, three patients will receive hAEC (0.5 × 10^6^/kg) as a single IV injection. If no SAE occur within 5 days of the last infusion in Cohort 1, a further three patients will be sequentially enrolled in Cohort 2 and receive a higher hAEC dose (1.0 × 10^6^/kg) as a single IV injection. If no SAE occur within 5 days of the last infusion in Cohort 2, a further three patients will be sequentially enrolled in Cohort 3 and receive the same hAEC dose as Cohort 2 (1.0 × 10^6^/kg) as a single IV injection at days 0 and 28. If no SAE occur within 5 days of the last infusion in Cohort 3, a further three patients will be sequentially enrolled in Cohort 4 and receive the same hAEC dose as Cohort 3 (1.0 × 10^6^/kg) as a single IV injection at days 0, 28, and 56 (**Table 1**).

**Table 1:** Indicative minimal timeline.

**Cohort**/patient number	**C1**/1, 2, 3 (One infusion/patient)	**C2**/4, 5, 6 (One infusion/patient)
Week of study	0–8	12–20
**Cohort**/patient number	**C3**/7, 8, 9 (Two infusions/patient)	**C4**/10, 11, 12 (Three infusions/patient)
Week of study	24–52	44–76


#### The Development of Clinically Significant Portal Hypertension

The natural history of fibrotic liver disease can be divided into stages that are relevant to survival and therefore to the development of therapeutic interventions. Some patients will have a cause for chronic liver injury that is amenable to therapy, for example, long-term antiviral therapy in chronic hepatitis B can control viral replication and therefore inhibit immune mediated liver injury, resulting in cirrhosis reversal. For patients with persistent liver injury for which no treatment is available or in whom treatment fails, fibrosis will progress. The onset of clinically significant portal venous hypertension is considered the ‘tipping point’ in this progression as patients who develop it are at risk of developing ascites, variceal hemorrhage, and porto-systemic shunting with hepatic encephalopathy – all events which define the progression from compensated to decompensated cirrhosis.

Measurement of the gradient between venous pressure in the hepatic vein and that in the portal vein [hepatic venous pressure gradient (HVPG)] is widely accepted as the most accurate way to define the severity of portal hypertension and thus the risk of developing death. An HVPG ≥ 10 defines clinically significant portal hypertension. Annual mortality rates increase from 1% in patients with compensated cirrhosis (HVPG 6–10 mmHg) to 3% in compensated cirrhosis with an HVPG ≥ 10 but ≤ 12 mmHg to 10–30% in decompensated cirrhosis (HVPG > 12 but ≤ 16 mmHg) or >60% in patients with very high portal pressures (>16 mmHg). Therefore portal pressure reduction is a therapeutic goal since a decrease in HVPG of ≥20% from baseline or to ≤12 mmHg reduces the risk of variceal bleeding and other complications of portal hypertension such as ascites (D’Amico et al., 2006).

#### Direct Measurement of Portal Pressure

The difference between free hepatic venous pressure (FHVP) (or inferior vena cava pressure) and wedged hepatic venous pressure (WHVP) defines HVPG. To measure the WHVP, the hepatic vein is catheterized using a balloon-tipped catheter under fluoroscopic control. The balloon is inflated to completely occlude a branch of the hepatic vein and the pressure (WHVP) is recorded. At that moment, the pressure measurement represents the hepatic sinusoidal pressure.

Hepatic venous pressure gradient measurement does require an experienced operator, often an interventional radiologist, and is an invasive procedure. However, the risks associated with the procedure are generally minor complications such as transient cardiac arrhythmias, local pain at puncture site, or vagal reaction and occur infrequently (<1% of patients) (Suk, 2014). One major laboratory reported no fatalities in over 12,000 studies (Bosch et al., 2009).

## Non-Invasive Methods to Measure Liver Fibrosis and Portal Hypertension

### Transient Elastography

Liver stiffness measurement (LSM) by vibration-controlled transient elastography (TE) is now in common clinical use and has supplanted liver biopsy as a method for routine assessment of liver fibrosis. At Monash Health, Fibroscan^TM^ (Echosens, Paris) is used for this purpose. TE involves transmitting a low amplitude vibration at low frequency to induce an elastic shear wave in the liver. The wave propagation speed is captured using pulse-echo ultrasonic acquisition and directly relates to tissue stiffness. This technology has been validated in patients with chronic viral hepatitis (HBV, HCV), non-alcoholic fatty liver disease and alcoholic liver disease with good correlation with histological liver fibrosis. TE is used most often to determine whether or not a patient has cirrhosis and overall has a high negative predictive value meaning that it is useful in excluding cirrhosis. Accurate detection of cirrhosis is greatest in patients with a pre-test probability of cirrhosis >75% and may vary depending on the etiology of cirrhosis. In regard to detecting portal hypertension, a recent study evaluated the use of Fibroscan in addition to HVPG, hepatic ultrasound appearance and platelet count to detect clinically significant portal hypertension and shown in a single center study that this may be possible (Augustin et al., 2014) however, LSM cannot currently replace direct techniques such as HVPG.

## Identification and Recruitment of Study Participants

Potential study participants will be identified and recruited from liver clinics staffed by the Monash Health Gastroenterology and Hepatology Unit. Schedule of events are summarized in **Table 2**.

**Table 2:** Schedule of events.

	Screening	Cell infusion				Follow up	
			
Cohort	All cohorts	1	2	3	4	All cohorts	
			
		Day 1	Day 1	Days 1, 30	Days 1, 30, 60	Day 5 post each infusion	Week 8 post last infusion
Informed consent	x						
Clinical assessment	x	x	x	x	x	x	x
Blood tests	x	x	x	x	x	x	
Serum sample	x	x	x	x	x	x	x
Chest X-ray	x						
HVPG	x						x
Ultrasound	x					x	x
Fibroscan	x					x	x
Biomarkers	x					x	x
Con meds	x	x	x	x	x	x	
hAEC infusion		x	x	x	x		


*Infusion days: observe in clinical trials unit for 24 h post infusion.*

## Inclusion Criteria

• Female or male
• Age ≥ 18 years, ≤ 70 years

Aetiology of liver disease, one or more of:

• Non-alcoholic fatty liver disease
• Alcohol related liver disease – abstinent for at least 3 months
• Hepatitis C virus infection (treated or not treated)
• Hepatitis B virus infection (on nucleoside analogs with normal ALT and HBV DNA) or inactive phase
• HIV co-infection with HCV/HBV with virological suppression > 12 months
• Cryptogenic cirrhosis
• Hemochromatosis

Cirrhosis, defined as one of:

• Liver biopsy confirming histological features of cirrhosis
• Transient elastography (Fibroscan) > 14.8 kPa
• FIB 4 > 3.25
• Clinical and radiological features that in the opinion of the investigator are consistent with a diagnosis of cirrhosis
• Compensated cirrhosis defined by HVPG 6–10 mmHg.

## Exclusion Criteria

• Current or previous episodes defining decompensated liver disease, including variceal hemorrhage, hepatic encephalopathy, ascites
• Listed for liver transplantation
• Active autoimmune disease (IgG > 2x ULN)
• Renal insufficiency (eGFR < 70 mL/min/1.73 m^2^)
• HIV infection (untreated or uncontrolled viraemia)
• HBV DNA > 200 IU/mL
• Fulminant hepatitis (acute severe hepatitis with encephalopathy)
• Primary sclerosing cholangitis
• Primary biliary cholangitis
• Portal/hepatic vein thrombosis
• Significant comorbidity (chronic heart failure, COAD, pulmonary hypertension, diabetes)
• Fibrotic liver disease other than cirrhosis (nodular regenerative hyperplasia)
• Inability or unwillingness to provide informed consent.

## Primary Endpoint

The primary objective of this pilot study is to establish the safety and tolerability profile of transplanted hAEC in adult patients with compensated cirrhosis.

## Secondary Objectives

The following secondary objectives will inform the development of future clinical trial protocols and enable appropriate statistical analysis

• Assess changes in baseline CPT, MELD, HVPG, liver stiffness and non-invasive tests at 3 and 6 months following cell exposure
• Assess health related quality of life
• Assess liver related clinical events.

## Design and Conduct of the Study

### hAEC Retrieval and Preparation

Healthy pregnant mothers delivering at term gestation will be approached and consented for donation of their amniotic membranes. The donor mothers will be screened for all viral and other infections (CMV, HIV, Hepatitis B and C virus – similar to blood transfusion donors) on day of delivery. These investigations will be repeated 3 months later again to exclude pathogens that might have been in incubation during delivery. hAECs will be isolated in a GMP-certified Biospherix Unit housed in the Monash Health Translation Precinct – Translational Research Facility (MHTP TRF) and stored in a secure and dedicated liquid nitrogen dewar until the donor has been cleared for use.

## Cell Harvest, Isolation, and Administration

### hAEC Isolation

Amniotic membranes will be collected from healthy women with a normal singleton pregnancy undergoing Cesarean section at term (37–40 weeks gestation). Informed written consent is obtained prior to surgery.

Human amnion epithelial cells are isolated from amnion membranes and purity is assessed as described previously (Murphy et al., 2014). Briefly, amniotic membranes are minced and digested twice for 60 min at 37°C using animal-product free recombinant trypsin (TrypZean, Sigma Aldrich) and re-suspended in animal-product free culture medium (EpiLife, Life Technologies) for cell counting. Purity is assessed by flow cytometry for EpCam+ cells (BD Biosciences) and batches that are >90% EpCam positive but negative for MSC markers (CD90, CD105) are cryopreserved in animal product free cryopreservation media (Cryostor CS5, Stem Cell Technologies).

## Monitoring

### Screening to Day of Treatment

Prior to first hAEC infusion, the following procedures will be performed:

• Obtain patient demographic information
• Physical examination and vital signs
• Baseline laboratory testing: full blood examination, liver function tests, INR, alpha fetoprotein, renal function tests, serum electrolytes, ESR, CRP, pregnancy testing in females
• Serum sample (10 mL) for storage
• Calculate CPT score and MELD score
• Transient elastography
• Performance status (ECOG)
• Chest X-ray.

### Monitoring on Day of Treatment

• Patients will be observed and monitored in the TRF Clinical Centre with appropriate monitoring facilities. Facilities to treat anaphylaxis are present and experienced trial personnel will be in attendance.
• Blood pressure and pulse oximetry monitoring every 15 min during the first 2 h, every 30 min for the next 2 h, every hour following that through to 24 h.
• Temperature measurement q30 min for 4 h and then hourly for first 12 h.
• Record any adverse events (as described below) and concomitant medications.
• Serum sample (10 mL) at 12 and 24 h for storage.

### Monitoring Days 2–5 Post hAEC Transplantation

The following will be performed after 24 h of therapy:

• Post infusion days 2–4: daily telephone contact to assess overall status
• Post infusion day 5: clinical assessment including open ended questioning to ascertain any adverse events, recording concomitant medications and measuring FBE, UEC, LFT, AST, CRP, INR. A 10 ml serum sample will be obtained for storage.
  Post infusion week 8 for cohorts 1 and 2, week 12 for cohort 3, week 16 for cohort 4: Routine laboratory tests (as for day 5), Fibroscan and HVPG measurements will be performed and a serum sample (10 ml) obtained for storage.

### Long-Term Follow-Up

All study subjects will be followed 6-monthly for a minimum of 2 years with abdominal ultrasounds and routine laboratory tests (FBE, LFT, UEC, INR, AFP). This testing regimen is the standard of care for cirrhotic patients who require lifelong surveillance for hepatocellular carcinoma.

## Outcomes

### Primary (Assessment of Safety)

(a) Local skin reactions – extravasation, erythema, edema.
(b) Anaphylaxis – acute deterioration of respiratory, cardiovascular parameters:
  Respiratory deterioration – dyspnoea, hypoxema, any requirement for supplemental oxygen or mechanical ventilation.
  Cardiovascular deterioration – hypotension, hypertension, bradycardia, tachycardia, rhythm abnormalities.
(c) Infection – clinical signs, laboratory evidence (increase in CRP, white cell counts, bacterial/viral growth on sterile cultures).
(d) Features of rejection as evidenced by impairment in hepatic or renal parameters.
  Hepatic – new onset jaundice, elevation of hepatic enzymes, coagulation dysfunction – thrombocytopenia, increase in INR.
  Renal – decrease in eGFR.

### Expected Outcomes

It is expected that the intravenous administration of hAECs in adults with compensated or decompensated cirrhosis will be generally safe. There is a theoretical risk of local and systemic side effects akin to those following blood transfusions. It is extremely unlikely that there will be development of rejection phenomena or long term tumor formation.

## Statistical Analysis

There is no planned formal statistical analysis of this phase I trial, but descriptive and inferential statistical analysis will be conducted as appropriate. Data emerging from the study will be used to develop statistical parameters for phases 2 and 3 studies. A data and safety monitoring board (DSMB) will be established to provide study oversight, peer review by an expert panel independent of the investigators to audit informed consent, research subject monitoring, study conduct, trial registration, and reporting.

## Consent

Informed written consent will be obtained from each patient who will retain the right to revoke consent at any time after inclusion in the trial without prejudice for future medical care.

## Data Collection and Analyses

Data will be collected prospectively and each individual patient will be monitored and outcomes reviewed following each administration of hAECs. If at any point, significant adverse events are noted directly or indirectly attributable to the hAECs, further recruitment of patients will be stopped pending review by the DSMB and investigators.

## Data Safety Monitoring

### Adverse Event Documentation

To protect the safety of the individual treated patients and others who may receive the drug in future, complete records of adverse events will be provided.

### Adverse Event Definition

An adverse event (AE) is any untoward medical occurrence in a patient administered a pharmaceutical product and that does not necessarily have a causal relationship with this treatment. An adverse event can therefore be any unfavorable and unintended sign (including an abnormal laboratory finding), symptom, or disease temporally associated with the use of a medicinal product, whether or not related to the medicinal product. Pre-existing conditions that worsen after drug administration will be reported as adverse events. Generally an increase/worsening of pre-existing disease status more than 30% of baseline is reported as an adverse event (for e.g., a baseline heart rate increase from 150/min to more than 195/min, an increase of FiO_2_ requirement from a baseline 50% to more than 65%).

### Adverse Event Severity and Relationship to hAEC

The severity of AEs will be graded using the National Cancer Institute Common Terminology Criteria for Adverse Events (NCI-CTCAE), v4.0. For AEs that are not identified in the NCI-CTCAE, the following scale will be used:

Grade 1: Mild

Grade 2: Moderate

Grade 3: Severe

Grade 4: Life-threatening or disabling

Grade 5: Death related to an AE.

**Causality assessment** will be made using the following definitions:

Unrelated:

This category is applicable to adverse events that are judged to be clearly and incontrovertibly due to extraneous causes (disease, environment, etc.) and do not meet the criteria for drug relationship listed under Unlikely, Possible or Probable.

Unlikely:

In general, this category is applicable to an adverse event that meets the following criteria (must have the first two):

(1) It does not follow a reasonable temporal sequence from administration of the drug.
(2) It may readily have been produced by the patient’s clinical state, environment or toxic factors, or other modes of therapy administered to the patient.
(3) It does not follow a known pattern of response to the suspected drug.
(4) It does not reappear or worsen if or when the drug is re-administered.

Possible:

This category applies to adverse events for which the connection with hAEC appears unlikely but cannot be ruled out with certainty. An adverse event may be considered possibly related if or when (must have the first two):

(1) It follows a reasonable temporal sequence from administration of the drug.
(2) It may have been produced by the patient’s clinical state, environment or toxic factors, or other modes of therapy administered to the patient.
(3) It follows a known pattern of response to the suspected drug.

Probable:

This category applies to adverse events that are considered with a high degree of certainty to be related to hAEC. An adverse event may be considered probable, if (must have the first three):

(1) It follows a reasonable temporal sequence from administration of the drug.
(2) It cannot be reasonably explained by the known characteristics of the patient’s clinical state, environment or toxic factors, or other modes of therapy administered to the patient.
(3) It disappears or decreases on cessation or reduction in dose.
(4) It follows a known pattern of response to the drug.
(5) It reappears on re-challenge.

### Serious Adverse Event Definition

A SAE is any untoward medical occurrence that occurs at any dose. An AE in a clinical trial is designated to be serious if it results in death, is life-threatening, requires inpatient hospitalization, or prolongs existing hospitalization, results in persistent or significant disability or incapacity.

### Serious Adverse Event Reporting

All SAEs that occur after hAEC administration (whether or not considered related to hAEC) will be reported within 24 h to the DSMB. Promptly after the occurrence of the event, the treating physician would submit an initial written report of any SAE to the HREC. Any necessary follow-up will be submitted within a reasonable time thereafter.

The independent DSMB will review each SAE report and evaluate the relationship of the SAE to hAEC and determine a need for further action. If the discovery of a new adverse event related to hAEC raises concern over the safety of continued administration of the drug, the DSMB and PI will take immediate steps to notify the regulatory authorities, HREC and all physicians who have treated patients with hAECs.

## Discussion

The purpose of this phase 1 trial is to investigate the safety of hAEC in patients with compensated cirrhosis. This study is significant because it will provide data that will inform the development of phases 2 and 3 clinical trials to test the safety and efficacy of cell therapy in patients whose only effective therapeutic option is whole organ transplantation. Liver transplantation is an established life-saving treatment for patients with end-stage liver disease; however, success depends on donor graft availability and recipients require life-long immunosuppression. hAEC based therapy overcomes both of these limitations due to widespread availability of the cells and the absence of a host immune response to the transplanted cells.

While the annual number of liver transplants performed in Australia has progressively increased, the number of donors has decreased. This has been associated with increased deaths on the transplant waiting list (Prakoso et al., 2010). In 2011, there were more than 500 patients on the Australia and New Zealand liver transplant waiting list, of which 10% were delisted due to death, becoming too ill or experiencing liver cancer progression (Lynch and Balderson, 2013). Although there are human clinical trials of stem/progenitor cell therapies that attempt to bridge this gap in supply and demand for donor organs, the mechanisms of action for such therapies are poorly understood and the outcomes have not translated into clinical practice. *Ex vivo* differentiation of embryonic stem cells into hepatocyte-like cells or transplantation of undifferentiated adult MSCs that differentiate into hepatocyte-like cells in animals with experimental liver injury have been reported (Sato et al., 2005; Basma et al., 2009). In models of chronic liver fibrosis, MSC transplantation has had varying results (Carvalho et al., 2008). Several issues complicate the clinical use of these cells such as tumor formation by ESC-derived hepatocytes (Basma et al., 2009), the negligible engraftment of MSC (Houlihan and Newsome, 2008), MSC fusion with host cells forming potential tumor cells and differentiation into cells that perpetuate hepatic fibrosis (Russo et al., 2006). As bone marrow derived mononuclear cell fractions includes diverse cell populations, it has been difficult to identify which subpopulation or cell interactions may be responsible for any changes in liver function.

The placenta, specifically the amniotic epithelium that develops from the epiblast prior to gastrulation, is an important source of stem cells. Amnion epithelial cells express embryonic stem cell markers but do not express telomerase and are non-tumorigenic (Murphy et al., 2010b). In addition to low immunogenicity, they have multi-lineage differentiation potential into cells derived from endoderm (liver, lung), mesoderm (bone, fat) and ectoderm (neural crest). Thus hAEC are pluripotent cells of fetal origin that have several advantages for the treatment of liver fibrosis. Compared to the 100s of 1000s of MSC that can be isolated from adipose tissue or bone marrow, 120–200 million hAEC are routinely isolated from a single term amnion using a method that *is free of animal products and compliant for clinical use* (Murphy et al., 2014). The isolation process for hAEC is approximately 4 h compared to MSC that require 4–6 weeks of *in vitro* expansion to obtain a similar number of cells for therapeutic use. hAEC have been transplanted into humans without evidence of rejection (Akle et al., 1981) even after 14-month follow up examination (Yeager et al., 1985). Furthermore, the collection and isolation of hAEC pose no ethical concerns as the placenta is normally discarded following delivery. Given our promising preclinical evidence, we expect that allogeneic hAECs will be well-tolerated.

We received approval from Monash Health for our human ethics application 16052A, “A Pilot Study Evaluating the Safety of Intravenously Administered Human Amnion Epithelial Cells for the Treatment of Liver Fibrosis, A First in Adult Human Study” on the 8th of August 2016. This trial has been registered on the Australian and New Zealand Clinical Trials Registry ACTRN12616000437460. The trial will be conducted in accordance to Monash Health Human Ethics guidelines and the Therapeutics Goods Administration was notified on the 30th of March 2017. Outcomes from this study will be disseminated in the form of conference presentations and submission to a peer reviewed journal.

## Ethics Statement

This study was carried out in accordance with the recommendations of Monash Health Human Research Ethics Committee with written informed consent from all subjects. All subjects gave written informed consent in accordance with the Declaration of Helsinki. The protocol was approved by the above mentioned Monash Health Human Research Ethics Committee.

## Author Contributions

All authors assisted with study protocol design, preparation of ethics application and drafting of this manuscript.

## Conflict of Interest Statement

The authors declare that the research was conducted in the absence of any commercial or financial relationships that could be construed as a potential conflict of interest.
